# HIV in Latin American migrants in the UK: A neglected population in the 95‐95‐95 targets

**DOI:** 10.1111/hiv.70007

**Published:** 2025-02-24

**Authors:** Natalie Elkheir, Catherine Dominic, Amy Price, Jessica Carter, Nadia Ahmed, David A. J. Moore

**Affiliations:** ^1^ Clinical Research Department London School of Hygiene and Tropical Medicine London UK; ^2^ Hospital for Tropical Diseases University College London Hospitals London UK; ^3^ UK Chagas Hub: https://www.uclh.nhs.uk/uk‐chagas‐hub; ^4^ Norfolk and Norwich University Hospitals NHS Trust Norwich UK; ^5^ London North West University Healthcare NHS Trust London UK; ^6^ City St George's University of London London UK; ^7^ Queen Mary's University of London London UK; ^8^ Genito‐Urinary Medicine Department Central and North West London NHS Foundation Trust London UK

**Keywords:** HIV, Latin America, migrant health, screening, underdiagnosis

## Abstract

**Background:**

The UK has reached the UNAIDS 2025 targets (of 95% of those living with HIV to be diagnosed, 95% to be on treatment and 95% of those on treatment to be virally suppressed). However, it is not known whether this target is met in subgroups such as migrant populations. Latin Americans are a fast‐growing migrant group in the UK and have low engagement with healthcare services, yet little is known about the HIV profile in this population. This study aimed to explore the profile of Latin American migrants under HIV care in the UK, and to identify any gaps in the 95‐95‐95 target in this population.

**Methods:**

Country of birth‐ and gender‐ stratified prevalence of HIV in Latin American migrants in the UK (as of December 2022) was estimated using data from the HIV and AIDS Reporting System and the Office for National Statistics. UNAIDS (2024) estimates from Latin American countries were used as proxies for expected prevalences and applied to Latin American demography in England to calculate expected cases. The ratio of the observed (or diagnosed) cases to the expected cases was calculated to estimate potential underdiagnosis.

**Results:**

A total of 2482 migrants born in Latin American countries were diagnosed with (and under care for) HIV in the UK (observed prevalence 841 per 100 000 population) by the end of 2022. The highest observed prevalence was in men born in Country 3 (2431 per 100 000 population) and the lowest in women born in Country 13 (30 per 100 000 population). Some 89% (*n* = 2219) of Latin American migrants under care for HIV were men, with 263 women under care with HIV in the whole of the UK. For women born in five of the countries of the region, it was estimated that only 14%, 17%, 25%, 26% and 33% of women living with HIV were under care.

**Conclusions:**

There may be a significant burden of underdiagnosis of HIV among Latin American women in the UK. Although based on some assumptions (notably the application of national HIV estimates to migrant populations which have a different risk profile), the gender disparity is striking. Community engagement and culturally appropriate targeted awareness raising and testing campaigns are recommended.

## INTRODUCTION

In 2023, the UK Health Security Agency (UKHSA) declared that the UK had reached the UNAIDS 2025 targets (of 95% of those living with HIV to be diagnosed, 95% to be on treatment and 95% of those on treatment to be virally suppressed) for the third year running [1]. UKHSA specifically reported that 95% were diagnosed, 98% of those diagnosed were on treatment and 98% of those on treatment were virally suppressed [2].

HIV diagnosis rates (among people first diagnosed in England) have decreased over time; however, a report from UKHSA in 2022 highlighted an increase in new HIV diagnoses among individuals born in South America, with this population accounting for a significant proportion of new diagnoses, and a notable rise compared to previous years [3, 4].

Migrant populations in the UK are disproportionately affected by HIV and are more likely to be diagnosed late compared to the general population. A report from the National AIDS Trust highlighted that 62% of new HIV diagnoses in 2019 were among migrants, mostly born in regions with high HIV prevalence [5]. Migrant populations face unique barriers to accessing healthcare, including language barriers, difficulty navigating new systems and precarious working conditions, which may all contribute to late diagnosis [6].

In the UK there are several vulnerable migrant populations in whom HIV underdiagnosis may be an issue. According to the 2021 Census, the UK is home to over 280 000 people born in Latin American countries, with over half resident in London [7]. Although Latin American migrants have arrived in the UK in large numbers since the 1970s, it was during the 1990s and 2000s that many continued to settle in the UK as students, refugees and economic migrants [8]. Migration was driven by economic stagnation and politic instability in Latin America, with restrictions on entering the United States following 9/11 also playing a part [8]. Secondary migration from other European Union (EU) countries began in the early 2000s, including many from Southern European countries affected by the economic recession in 2008 [8]. The Latin American community now represents London's second fastest growing migrant population from outside the EU, after the Chinese population [8]. A British HIV Association (BHIVA) report by Rawson et al. noted that, between 2008 and 2017, the Latin American migrant population constituted 3.8% of all new HIV diagnoses in the UK [9]. Additionally, the number of new diagnoses in this population increased over the same period, and a higher proportion of individuals were diagnosed late compared to the general population, notably among heterosexual men and women compared to men who have sex with men (MSM).

It is challenging to estimate whether the 95‐95‐95 target is truly being met in migrant populations. Most evidence on HIV diagnosis in migrants in the UK refer to those born in sub‐Saharan Africa, while the profile of HIV diagnosis in Latin American migrants living in Europe remains relatively unexplored. This study aimed to address this knowledge gap, to explore the profile of HIV diagnoses in Latin American migrants in the UK, and to identify any gaps in the 95‐95‐95 target in this population.

## METHODS

This was a cross‐sectional study, utilizing secondary data analysis of routinely collected sources of data.

### Population definition

The definition of Latin American migrant used in this study was people born in Spanish‐ and Portuguese‐speaking countries of Mexico, Central America, South America and the Caribbean islands, which is the definition provided in both McIlwaine's ‘Towards Visibility’ report [8] and the UNAIDS 2024 data report [10]. This includes 19 countries: Mexico, Guatemala, El Salvador, Honduras, Nicaragua, Costa Rica, Panama, Colombia, Venezuela, Ecuador, Peru, Brazil, Bolivia, Paraguay, Argentina, Uruguay, Chile, Cuba and the Dominican Republic. To avoid any risk of patient identification, country names have been replaced with Country 1, 2, 3 (etc.) throughout the article.

### Data sources

#### Individuals seen for HIV care in the UK

Data on individuals seen for HIV care in the UK were prepared and supplied by the Blood Safety, Hepatitis, Sexually Transmitted Infections (STI) and HIV division at the UK Health Security Agency (UKHSA) at the study authors' request. The data were derived from the HIV and AIDS Reporting System (HARS) (up to the end of December 2022) and HIV and AIDS New Diagnoses Database (HANDD) [11] (January–December 2022). All gender data refer to gender identity, as reported by the HIV clinic. Men includes transgender (trans) men, and women includes transgender (trans) women. Where gender identity information was not reported, gender at birth was used. Patient counts fewer than five in any given group were concealed to protect confidentiality. Site of diagnosis was also obtained from the HANDD database, but was only available for the year 2019.

#### Latin American migrant population sizes

Latin American migrant population sizes were derived from the Office for National Statistics' (ONS) ‘Population of the UK by individual country of birth and nationality: July 2020 to June 2021’ database [12]. This database was stratified by country of birth, but not gender. Gender‐stratified population sizes were estimated using the assumption of a men:women ratio of 45:55 as per McIlwaine et al.'s report on the gender structure of the Latin American community in London [8].

#### Estimated HIV prevalence

Estimated HIV prevalences, stratified by country of birth and gender, were derived from the Latin American country profiles of the UNAIDS Data Report 2024 [10].

### Data analysis

The number of diagnosed cases of HIV was divided by the overall population of migrants as estimated by the ONS to obtain an observed prevalence (overall and stratified by country of birth and gender).

Expected country‐specific prevalences for each migrant group were calculated based on gender‐stratified HIV prevalence estimates (from each country of birth in Latin America) in the UNAIDS 2024 dataset.

To estimate the burden of undetected HIV in the Latin American migrant population in the UK, the ratio between observed and expected prevalence was calculated (number of diagnosed cases divided by number of estimated cases).

All analyses were conducted using Microsoft Excel and STATA version 18.

## RESULTS

### Overall profile of HIV

According to the HIV and AIDS Reporting System, there were 2482 migrants born in Latin American countries under care for HIV in the UK (observed prevalence 841 per 100 000 population) at the end of 2022. Table 1 outlines the profile of individuals seen for HIV care, stratified by country of birth and gender.

**TABLE 1:** Profile of Latin American migrants with a diagnosis of HIV in the UK, 2022.

Country of birth	Men^a^					Women^a^				All
	Cases, *n*	Mean age, years	On ART, *n* (%)	HIV care in London, *n* (%)	MSM, *n* (%)	Cases, *n*	Mean age, years	On ART, *n* (%)	HIV care in London, *n* (%)	Cases, *n*
Country 1	113	46	112 (99)	74 (65)	96 (85)	7	48	7 (100)	<5	120
Country 2	21	41	20 (95)	15 (71)	17 (81)	5	37	<5	<5	26
Country 3	1302	42	1285 (99)	974 (75)	1149 (88)	153	44	150 (98)	96 (63)	1455
Country 4	37	43	37 (100)	17 (46)	34 (92)	<5	57	<5	0 (0)	40
Country 5	276	46	275 (99.6)	223 (81)	251 (91)	22	52	22 (100)	17 (77)	298
Country 6	6	37	6 (100)	<5	6 (100)	0	–	–	–	6
Country 7	30	48	30 (100)	18 (60)	27 (90)	<5	39	<5	<5	33
Country 8	23	47	22 (96)	14 (61)	14 (61)	23	43	22 (96)	16 (70)	46
Country 9	65	45	64 (98)	59 (91)	52 (80)	13	47	13 (100)	11 (85)	78
Country 10	26	37	26 (100)	13 (50)	23 (88)	0	–	–	–	26
Country 11	14	37	14 (100)	8 (57)	13 (93)	<5	60	<5	<5	17
Country 12	16	33	16 (100)	9 (56)	14 (88)	<5	47	<5	<5	19
Country 13	80	41	78 (98)	47 (59)	73 (91)	<5	47	<5	0 (0)	83
Country 14	5	53	5 (100)	<5	5 (100)	0	–	–	–	5
Country 15	12	45	12 (100)	5 (42)	11 (92)	<5	58	<5	<5	15
Country 16	9	44	9 (100)	8 (89)	9 (100)	<5	44	<5	<5	12
Country 17	59	45	57 (97)	35 (59)	52 (88)	7	49	6 (86)	<5	66
Country 18	9	52	9 (100)	6 (67)	7 (78)	<5	52	<5	0 (0)	12
Country 19	116	43	116 (100)	61 (53)	103 (89)	9	42	9 (100)	7 (78)	125
Total	2219	44	2193 (99)	1592 (72)	1956 (88)	263	48	256 (97)	171 (65)	2482

*Note*: Data prepared and supplied by Blood Safety, Hepatitis, Sexually Transmitted Infections (STI) and HIV Division at the UK Health Security Agency (UKHSA) at study authors' request. Data source: HIV and AIDS Reporting System (HARS) and HIV and AIDS New Diagnosis and Deaths (HANDD) ‐ data to the end of December 2022. Numbers fewer than five (which were concealed to protect confidentiality) have been rounded to three for the total calculations.

Abbreviations: ART antiretroviral therapy; MSM, men who have sex with men.

^a^ Gender: All gender data refer to gender identity, as reported by the clinic. Full reporting of gender identity began in 2015. Men includes transgender (trans) men, and women includes transgender (trans) women. Where gender identity information is not reported, gender at birth is used in the table.

The majority (59%) of Latin American migrant people living with HIV in the UK were from Country 3 (*n* = 1455), followed by Country 5 (*n* = 298, 12%), Country 19 (*n* = 125, 5%), Country 1 (*n* = 120, 5%) and Country 9 (*n* = 78, 3%,) (Table 1). Of men diagnosed with HIV, 88% (*n* = 1956) were MSM (Table 1).

### HIV profile by gender

The highest observed prevalence was in men born in Country 3 (2431 per 100 000 population) and the lowest in women born in Country 13 (30 per 100 000 population). Some 89% (*n* = 2219) of Latin American migrants diagnosed with HIV were men, with 263 women under care for HIV in the whole of the UK.

Between January and December 2022, 205 new diagnoses of HIV were made in Latin American migrants, 84% of whom were men (Table 2). The mean age at diagnosis was 36 years in men and 39 years in women.

**TABLE 2:** New diagnoses of HIV in Latin American migrants in the UK, January–December 2022.

Country of birth	Men^a^					Women^a^				
	New diagnoses, *n*	Mean age at diagnosis, years	Mean CD4, cells/mm^3^	CD4 < 200 cells/mm^3^, *n*	Mean viral load	New diagnoses, *n*	Mean age at diagnosis, years	Mean CD4, cells/mm^3^	CD4 < 200 cells/mm^3^, *n*	Mean viral load
Country 1	5	40	591	[x]	51	0	–	–	–	–
Country 2	3	38	362	[x]	25 118	<5	28	533	[x]	0
Country 3	95	32	584	7	65 136	21	39	703	<5	152 853
Country 4	<5	35	209	[x]	15 800	0	–	–	–	–
Country 5	13	38	479	<5	79 633	0	–	–	–	–
Country 6	<5	31	707	[x]	34	0	–	–	–	–
Country 7	<5	41	318	<5	282 400	0	–	–	–	–
Country 8	<5	34	775	[x]	[x]	<5	48	242	<5	320 000
Country 9	<5	44	116	<5	[x]	<5	33	760	[x]	0
Country 10	5	31	484	<5	25	0	–	–	–	–
Country 11	0	‐	‐	‐	‐	**0**	–	–	–	–
Country 12	9	28	510	<5	62 966	0	–	–	–	–
Country 13	12	36	583	[x]	53 236	<5	46	571	[x]	45 644
Country 14	<5	27	521	[x]	23 200	0	–	–	–	–
Country 15	<5	35	[x]	[x]	[x]	0	–	–	–	–
Country 16	0					0	–	–	–	–
Country 17	<5	42	519	<5	78 949	0	–	–	–	–
Country 18	<5	50	554	[x]	10	0	–	–	–	–
Country 19	<5	33	446	[x]	16 200	0	–	–	–	–
Total	172	36	485	14	50 197	33	39	562	<5	103 699

*Note*: Data prepared and supplied by Blood Safety, Hepatitis, Sexually Transmitted Infections (STI) and HIV Division at the UK Health Security Agency (UKHSA) at the study authors' request. Data source: HIV and AIDS Reporting System (HARS) and HIV and AIDS New Diagnosis and Deaths (HANDD) – data to the end of December 2022. Numbers less than five were concealed to protect confidentiality. [x] – data unavailable.

^a^ Gender: All gender data refer to gender identity, as reported by the clinic. Full reporting of gender identity began in 2015. Men includes transgender (trans) men, and women includes transgender (trans) women. Where gender identity information is not reported, gender at birth is used in the table.

In men, the observed prevalences of HIV were greater than the expected prevalences (based on UNAIDS birth country estimates) in all countries except Country 2, Country 4, and Country 18 (Table 3).

**TABLE 3:** Diagnosed and expected prevalence of HIV in Latin American migrants in the UK.

Country of birth	Men^a^				Women^a^			
	Population men in UK, *n* ^b^	Diagnosed HIV cases, n (prevalence %)^c^	Expected HIV cases, *n* (prevalence %)^d^	Diagnosed/expected cases^e^	Population women in UK, *n* ^b^	Diagnosed HIV cases, *n* (prevalence %)^c^	Expected HIV cases, *n* (prevalence %)^d^	Diagnosed/expected cases^e^
Country 1	11 700	113 (0.97)	57 (0.49)	1.97	14 300	7 (0.05)	40 (0.28)	0.17
Country 2	4050	21 (0.52)	23 (0.57)	0.91	4950	5 (0.1)	11 (0.21)	0.47
Country 3	53 550	1302 (2.43)	[x]	[x]	65 450	153 (0.23)	[x]	[x]
Country 4	4500	37 (0.82)	53 (1.18)	0.69	5500	<5 (<0.09)	12 (0.22)	0.25
Country 5	16 650	276 (1.66)	165 (0.99)	1.67	20 350	22 (0.11)	43 (0.21)	0.51
Country 6	[x]	6 ([x])	[x] (0.84)	[x]	[x]	0 (0)	[x] (0.14)	[x]
Country 7	2700	30 (1.11)	26 (0.95)	1.16	3300	<5 (<0.15)	7 (0.22)	0.43
Country 8	1800	23 (1.28)	18 (1)	1.28	2200	23 (1.05)	20 (0.93)	1.13
Country 9	9450	65 (0.69)	49 (0.52)	1.33	11 550	13 (0.11)	31 (0.27)	0.42
Country 10	[x]	26 ([x])	[x] (0.61)	[x]	[x]	0 (0)	[x] (0.34)	[x]
Country 11	900	14 (1.56)	2 (0.23)	6.81	1100	<5 (<0.45)	2 (0.15)	1.5
Country 12	[x]	16 ([x])	[x] (0.22)	[x]	[x]	5 ([x])	[x] (0.15)	[x]
Country 13	11 700	80 (0.68)	76 (0.65)	1.05	14 300	<5 (<0.03)	21 (0.15)	0.14
Country 14	[x]	5 ([x])	[x] (0.39)	[x]	[x]	0 (0)	[x] (0.18)	[x]
Country 15	[x]	12 ([x])	[x] (1.47%)	[x]	[x]	<5 ([x])	[x] (0.53%)	[x]
Country 16	900	9 (1)	6 (0.68)	1.47	1100	<5 (<0.45)	4 (0.32)	0.75
Country 17	2700	59 (2.19)	21 (0.78)	2.80	3300	7 (0.21)	8 (0.23)	0.93
Country 18	1800	9 (0.50)	14 (0.75)	0.67	2200	<5 (<0.23)	9 (0.41)	0.33
Country 19	10 350	116 (1.12)	73 (0.71)	1.58	12 650	9 (0.07)	34 (0.27)	0.26
Total	132 750	2219 (1.67)	–	–	162 250	263 (0.16)	–	–

*Note*: [x] – data unavailable (eg, in some countries no gender‐stratified prevalence estimate was available from UNAIDS).

^a^ Gender: All gender data refer to gender as defined in the original source data.

^b^ UK Latin American population sizes obtained from the Office for National Statistics (ONS) ‘Population of the UK by individual country of birth and nationality: July 2020 to June 2021’. A men:women ratio of 44:55 was assumed as per McIlwaine and Bunge [8]. Where fewer than three people with the relevant characteristics were sampled, the ONS does not supply an estimate due to disclosure control and this is indicated as [x] in the table.

^c^ Data prepared and supplied by Blood Safety, Hepatitis, Sexually Transmitted Infections (STI) and HIV Division at the UK Health Security Agency (UKHSA) at study authors' request. Data source: HIV and AIDS Reporting System (HARS) and HIV and AIDS New Diagnosis and Deaths (HANDD) ‐ data to the end of December 2022. Numbers fewer than five were concealed to protect confidentiality and rounded to three for calculations.

^d^ Expected prevalence was obtained from the UNAIDS Data 2024 Latin American country profiles.

^e^ Observed (diagnosed) cases divided by expected cases.

In women, however, the observed prevalences were generally lower than the expected prevalences. For example, for women born in Country 13, Country 1, Country 4, Country 19 and Country 18, it was estimated that only 14%, 17%, 25%, 26% and 33%, respectively, of women living with HIV were under care in the UK (Table 3).

### Diagnosis and HIV care

Of newly diagnosed Latin American patients in 2022, fewer than 18 (<10%) patients had a CD4 count of less than 200 cells per mm^3^ at diagnosis (classified as very late diagnosis, exact number suppressed to protect patient confidentiality).

**FIGURE 1:**
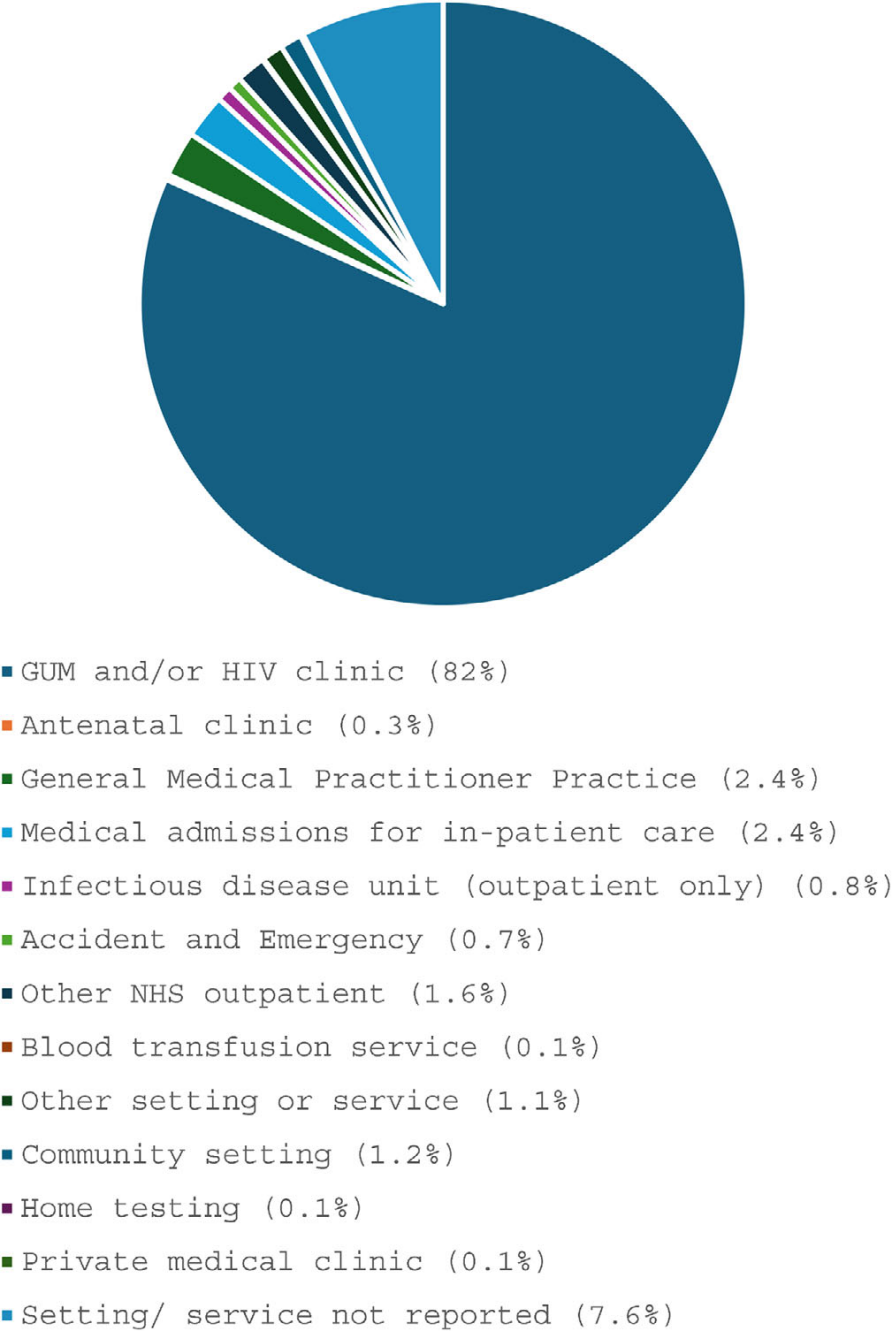
Setting of HIV diagnosis for Latin American migrants in the UK, 2019. Data source: HIV and AIDS Reporting System (HARS) – reports received to end of 2019. GUM, genitourinary medicine; NHS, National Health Service.

In the year 2019, 82% of cases were diagnosed in genitourinary medicine (GUM)/sexual health or HIV clinics, 2% in general practice and 2% during medical admission for inpatient care (Figure 1).

A total of 1763 (71%) of Latin American people living with HIV in the UK receive HIV care in London; and 2449 (99%) were on antiretroviral therapy (ART) (Table 1).

## DISCUSSION

This article reports a secondary data analysis of multiple sources of routinely collected data on the diagnosis of HIV in the Latin American migrant population in the UK. This analysis suggests a high prevalence of HIV among Latin American men in the UK (an overall prevalence of diagnosed cases of HIV of 1671 per 100 000, compared with an estimated 212 per 100 000 for men overall in the UK) [2], but highlights potential underdiagnosis in Latin American women migrants (in whom the diagnosed prevalence was 162 per 100 000).

According to the International Organization for Migration, 281 million people (4% of the world's population) were international migrants in 2020, of whom 135 million were women [13]. In addition to the usual inequalities migrants face, such as barriers to healthcare access due to migration status, women migrants may face a compounded inequality due to sociocultural influence, financial constraints and stigma. These factors mean migrant women may be especially vulnerable to HIV infection during their time of migration [14].

This study signalled significant underdiagnosis in Latin American migrant women in the UK. At World Health Organization European Region level in 2018, 19% of new HIV diagnoses were among migrant women originating from outside of Europe [15]. A cross‐sectional study on HIV diagnosis in migrant populations across nine countries in Western Europe (Belgium, Germany, Greece, Italy, the Netherlands, Portugal, Spain, Switzerland and the UK) between 2013 and 2015 found that 60% of women migrants presented with a late diagnosis, despite over 70% having previously accessed health services [16]. Additionally, 50% of women who had accessed antenatal care reported not having had a previous HIV test, again indicating substantial missed opportunities for earlier diagnosis. Among women, the reported barriers to accessing care included long waiting times in clinics, not trusting general practitioner (GP) confidentiality, and being unsure of one's right to access care. It is an interesting finding in this article that despite a signal of underdiagnosis of HIV in Latin American women migrants, there was a lack of evidence pointing towards late diagnosis in this population (in new diagnoses in 2022). In fact, very few women presented with CD4 counts lower that 200 cells/mm^3^. It is beyond the scope of this exploratory analysis to explain this phenomenon. However, to further understand the epidemiology of HIV in the Latin American migrant community, future research is recommended which should take into consideration variables (which were not available in this analysis) such as age stratification, HIV transmission routes and socioeconomic factors such as employment, housing and language.

A study published in 2015 of HIV cases reported to the European Surveillance System also correlates with our findings as regards HIV in the Latin American migrant population [17]. Of 156 817 HIV cases reported in Europe, 60 446 (38%) were migrants and 12% were from Latin America. The number of HIV cases from Latin America peaked in 2010 and has decreased thereafter. This trend was not observed in other migrant groups, and is likely explained by changes in migration patterns rather than prevention efforts. Further, both men and women migrants from Latin America had higher odds of late HIV presentation than native men and women. The proportion of late diagnosis among women migrants from Latin America in Europe was also higher than that seen in male migrants from Latin America, of whom 43% presented with a late diagnosis. Late diagnosis in women indicates that gender‐sensitive counselling and testing, including information about broader sexual and reproductive health needs, is needed in this population [18].

In McIlwaine et al.'s ‘Towards Visibility’ report, the socioeconomic determinants of health for the UK's Latin American migrants – which must be understood to address the potential of underdiagnosis of HIV in this community – are well described [8]. A quarter of this population work in low‐paid elementary jobs and a further 20% in other low‐paid sectors, with 20% also reporting poor English language skills. Although 90% of onward Latin American migrants (those who have moved from mainland Europe to the UK) reported having used the National Health Service (NHS) for themselves or their families, one in six were not registered with a GP [8, 19]. This is a cause for concern, as GPs are the main point of contact for access to all other health services in the UK, and the data on setting of HIV diagnosis presented in this article (Figure 1) suggest GPs are being underutilized by this population. The healthy migrant effect could also explain the underutilization of primary care by Latin American migrants. Notably, HIV testing in primary care in the UK in all populations increased between 2000 to 2010 but has been declining since 2010 [20]. Universal access to primary care is an essential basis for the NHS's current efforts in HIV testing, treatment and prevention. The lack of registration of Latin American migrants with GPs may mean the population remains particularly at risk of underdiagnosis, and there is a dearth of information regarding the population's engagement with sexual health services.

Research outside the UK has also cited the hostile healthcare environment to be a factor that might hinder access to HIV testing, and increase the risk of late diagnosis [21]. For example, migrants may experience specific legal and administrative impediments to accessing HIV testing, as well as cultural and linguistic barriers, alongside systemic racism and xenophobia [22]. A study of Latin American migrant women in Canada showed that women's vulnerability to HIV and sexually transmitted infections (STIs) was determined by experiences during their life course and their migratory status, including associations with sexual abuse, abuse at work, language barriers, lack of social support networks, and their ability to access health services [23].

Increasing HIV testing coverage, particularly through targeted campaigns, is a means of tackling health inequalities, given that HIV disproportionately affects minority groups [24]. Community engagement and peer education, and working with existing community groups and charities that have longstanding relationships with the community, can be useful for increasing access to HIV diagnostic and treatment services in migrant populations [25]. Furthermore, targeted awareness‐raising campaigns – including delivery in Spanish and Portuguese to target the Latin American community in the UK – should be developed. Finally, working to remove the legal, cultural and administrative barriers to HIV testing for migrants will aid earlier diagnosis and treatment of these vulnerable populations.

There are many limitations to this study to be considered. Primarily, the application of national HIV estimates from countries of origin to migrant populations for calculation of expected rates may be inaccurate, since migrant populations have a different HIV risk profile. Second, other migrant populations (for whom there is also limited published data) were not evaluated, and this may have revealed a gender disparity across all migrant groups. Some country‐specific analyses were based on very low case numbers, resulting in potential statistical instability of these findings. Furthermore, this study was based on registered data on country of birth and thereby systematically ignored second‐generation migrants who, again, may have a different risk profile. Additionally, a proportion of Latin American women who have received antenatal care in the UK are likely to have been routinely screened for HIV; however, no testing data were assessed in this study. It should be noted that the UKHSA data on individuals under HIV care in the UK was reported up until the end of 2022, and so includes time during the COVID‐19 pandemic. It is therefore possible that fewer people were engaged in care due to pandemic‐related healthcare access issues, rather than specific concerns relating to the Latin American community. Additionally, the data sources used in this analysis were not all available for the same year (eg, the UK HIV prevalence data is from 2022, whereas the Latin American migrant population size is from 2021). This is appropriate for this exploratory analysis, but further work is recommended to understand the temporal trends more thoroughly. Although the focus of the discussion has been narrowed to underdiagnosis of HIV among women Latin American migrants, it should be noted that underdiagnosis could still be a feature among men (and, if so, the absolute undiagnosed case numbers would be greater overall). It is a further limitation that expected (gender‐stratified) prevalence estimates were not available from the UNAIDS data source for Country 3, despite these citizens comprising the largest Latin American migrant group in the UK. Finally, it is a limitation of this study that data on sex work (among both men and women migrants with HIV) were not available and it is recommended that this factor be examined. Despite these limitations, the interrogation of the data presented in this article signals underdiagnosis of HIV among Latin American migrant women in the UK and warrants further investigation.

## CONCLUSIONS

This study has highlighted a potentially significant burden of underdiagnosis of HIV in Latin American women migrants in the UK, concluded from comparison of observed and expected HIV prevalence estimates. Although based on some assumptions, the gender disparity is striking. Community engagement and subsequent targeted awareness raising and testing campaigns are crucial to addressing any gaps in diagnosis and treatment. Further research and engagement with the community is needed to support where and how such a campaign (through primary care or sexual health services, for example) would be most impactful.

## AUTHOR CONTRIBUTIONS

Conceptualization: NE, NA, DAJM. Data curation: NE, CD. Formal analysis: NE. Methodology: NE, JC, NA, DAJM. Writing – original draft: NE, CD, AP. Writing – review and editing: NE, CD, AP, JC, NA, DAJM.

## FUNDING INFORMATION

NE is supported by a Clinical Research Fellowship from the UK Medical Research Council (MRC). JC is supported by a Wellcome Trust PhD Programme for Primary Care Clinicians Award.

## CONFLICT OF INTEREST STATEMENT

No conflicts of interest declared.

## ETHICS STATEMENT

This article reports on secondary data analyses of publicly available data sources and, as such, no specific ethical approval was sought.

## Data Availability

The data supporting the findings of this article appear within the article.
